# *Drosophila* Torsin Protein Regulates Motor Control and Stress Sensitivity and Forms a Complex with Fragile-X Mental Retardation Protein

**DOI:** 10.1155/2016/6762086

**Published:** 2016-05-30

**Authors:** Phuong Nguyen, Jong Bok Seo, Hyo-Min Ahn, Young Ho Koh

**Affiliations:** ^1^Department of Biomedical Gerontology, Graduate School of Hallym University, Chuncheon, Gangwon-do 24252, Republic of Korea; ^2^Ilsong Institute of Life Science, Hallym University, Anyang, Gyeonggi-do 14066, Republic of Korea; ^3^Korea Basic Science Institute, Seongbuk-gu, Seoul 02841, Republic of Korea

## Abstract

We investigated unknown* in vivo* functions of Torsin by using* Drosophila* as a model. Downregulation of* Drosophila* Torsin (DTor) by DTor-specific inhibitory double-stranded RNA (RNAi) induced abnormal locomotor behavior and increased susceptibility to H_2_O_2_. In addition, altered expression of DTor significantly increased the numbers of synaptic boutons. One important biochemical consequence of DTor-RNAi expression in fly brains was upregulation of alcohol dehydrogenase (ADH). Altered expression of ADH has also been reported in* Drosophila* Fragile-X mental retardation protein (DFMRP) mutant flies. Interestingly, expression of DFMRP was altered in DTor mutant flies, and DTor and DFMRP were present in the same protein complexes. In addition, DTor and DFMRP immunoreactivities were partially colocalized in several cellular organelles in larval muscles. Furthermore, there were no significant differences between synaptic morphologies of* dfmrp* null mutants and* dfmrp* mutants expressing DTor-RNAi. Taken together, our evidences suggested that DTor and DFMRP might be present in the same signaling pathway regulating synaptic plasticity. In addition, we also found that human Torsin1A and human FMRP were present in the same protein complexes, suggesting that this phenomenon is evolutionarily conserved.

## 1. Introduction

Dystonia is a phenomenological disorder encompassing various degrees of involuntary muscle contractions often initiated or worsened by voluntary actions [1]. Even though there are many different forms of heritable dystonia, Dystonia 1 (DYT1) dystonia has unique clinical characteristics such as early age of onset accompanied by generalized manifestation and progression of symptoms [1]. DYT1 dystonia is mainly caused by a 3 bp in-frame deletion in* TOR1A* resulting in a loss of glutamic acid (ΔE) in Tor1A [2]. Among humans with heterozygous Tor1A^ΔE^ mutations, only ~30% of them manifested severe symptoms in their adolescence, and the patient's life expectancy was not affected [3]. In addition, patients with homozygous Tor1A^ΔE^ mutations have not yet been reported [2, 3]. These inheritance patterns of DYT1 dystonia suggest that complete loss of Tor1A function may cause lethality during embryogenesis and that heterozygous Tor1A^ΔE^ mutation may increase susceptibility to other genetic or environmental risk factors that caused severe symptoms [4, 5].

To understand both the molecular and cellular basis of this disorder and the* in vivo* functions of Tor1A, various animal models have been generated. Because homozygous DYT1 global knock-in (Tor1A^ΔE/ΔE^) or global knockout (Tor1A^−/−^) mice died shortly after birth [6], homozygous mutant mice could not be used to study the abnormal behaviors. To overcome these problems, a mouse model in which Tor1A was knocked out specifically from the central nervous system was generated and showed severe dystonic symptoms even though those mice died within 16 days after birth [7].

Other mouse models generated by overexpressing Tor1A^ΔE^ in neurons showed very weak behavioral symptoms such as defects in beam walking [4], motor learning [8, 9], or paw-print analysis [9, 10]. Heterozygous knock-in mutant Tor1A^(ΔE/+)^ mice exhibited defects in beam-walking test and hyperactivity in open field test [11]. These results suggested that Tor1A^ΔE^ alone may be sufficient to alter neuronal circuits to cause mild behavioral defects, but not sufficient to evoke severe dystonic symptoms. To explain this possibility, the second hit models were suggested [5, 8].

*Drosophila* models provide unique opportunities to investigate the cellular and molecular etiologies underlying various neurological disorders in humans [12]. In a previous study, we generated a* Drosophila* model of DYT1 dystonia by expressing human Tor1A^ΔE^  (HTor1A^ΔE^) [5]. We found that expression of Tor1A^ΔE^ induced formation of protein aggregates and abnormalities at synapses and nuclear membranes. In addition, dystonic symptoms were observed when additional environmental stress was present. Furthermore, we found that expression of Tor1A^ΔE^ activated unfolded protein responses and upregulated heat shock protein 22 to compensate for the toxic effects of mutant Tor1A proteins [13]. However, the* in vivo* function of* Drosophila* Torsin (DTor) in the maintenance and regulation of locomotor control and synaptic plasticity is still not clear.

Maintenance of normal motor control is one of the most important factors for the survival and development of animals. Recent studies have suggested that proper motor control is regulated by many different types of neural circuits in central and peripheral nervous systems acting in harmony [14]. Mutations that cause severe defects in motor control in humans are known to cause various defects in nervous systems [15]. To investigate the* in vivo* functions of Torsin proteins, we generated transgenic flies expressing full-length DTor-cDNA or DTor-RNAi constructs. We performed various experiments to elucidate behavioral, biochemical, physiological, and proteomic consequences of altered Torsin protein levels in* Drosophila*. We found that Torsin proteins formed complexes with Fragile-X mental retardation protein (FMRP) in* Drosophila* and humans, and the neuromuscular junction (NMJ) morphology of* Drosophila fmrp* (*dfmrp*) mutant larvae expressing DTor-RNAi was similar to that of* dfmrp* mutant larvae, suggesting possible involvement of FMRP in DYT1 dystonia in regulation of motor control and synaptic plasticity.

## 2. Materials and Methods

### 2.1. Drosophila Genetics

*Drosophila* used in this study were raised at 25 ± 1°C with 60% relative humidity. The open reading frame of* Drosophila* Torsin (DTor) was amplified from a* Drosophila* cDNA library and verified by sequencing both strands of cloned DNA. The DTor-cDNA was subcloned into pUAST germ line transfer vectors. The first 500 bp of the 1st exon and intron of DTor genomic DNA was amplified, cloned, and subcloned into pWIZ germ line transformation vectors designed to express double-stranded inhibitory RNA [18] after verifying its sequence. Transgenic flies were generated using typical germ line transfer techniques. Additional RNAi flies used in this study were obtained from the Vienna* Drosophila* RNAi Center (Stock No. 30985, VDRC, Vienna Biocenter Core Facilities, Vienna, Austria). Previously characterized* UAS-HTor1A* ^*WT*^ *, Tubulin-Gal4* (Tub-Gal4),* C155-Gal4*, and* C57-Gal4* flies were used [5, 19].* dfmrp* null mutants,* fmr1* ^Δ*50M*^/*TM6b, Tb* ^*1*^ were obtained from the Bloomington* Drosophila* Stock Center (Indiana University, Bloomington, IN, USA).* fmr1* ^Δ*50M*^ and Tub-Gal4 chromosomes were recombined to generate* dfmr1* ^Δ*50M*^,* Tub-Gal4/TM6b, Tb* ^*1*^.* pWiz-DTor-RNAi*;* Tub-Gal4/TM6b, Tb* ^*1*^ flies were generated by recombining the 2nd and 3rd chromosomes.

### 2.2. Prediction of Glycosylation in DTor

The possible glycosylation patterns of DTor proteins were predicted using NetNGlyc 1.0 server (http://www.cbs.dtu.dk/services/NetNGlyc/) for N-glycosylation, the NetOGlyc 4.0 server (http://www.cbs.dtu.dk/services/NetOGlyc/) for O-glycosylation [20], and the NetCGlyc 1.0 sever (http://www.cbs.dtu.dk/services/NetCGlyc/) for C-glycosylation [21]. Only a single asparagine residue in DTor was predicted to be N-glycosylated (Supplementary Figure 1 in Supplementary Material available online at http://dx.doi.org/10.1155/2016/6762086).

### 2.3. Antibodies and Immunohistochemistry

To produce antibodies for DTor, His-tagged DTor recombinant peptides (residues 25–210) were expressed in* Escherichia coli* M15 carrying the pREP4 plasmid and then purified using Ni-NTA beads (Qiagen, Valencia, CA, USA). After SDS-PAGE separation, the fusion proteins were injected into rabbits for antibody generation.

The procedure for immunocytochemistry was as described in Koh et al. [5]. Briefly, the number of synaptic boutons in neuromuscular junctions (NMJs) in larval muscles 6 and 7 (abdominal segment 2) from experimental and control larvae was examined under epi-fluorescence microscope. Using Minitab software (Minitab Inc., State College, PA, USA), two-sample *t*-tests were used to determine significant differences. The antibodies used were anti-HRP-FITC (Jackson ImmunoResearch Laboratory Inc. West Grove, PA, USA; 4% paraformaldehyde in 0.1 M PB, 1 : 100), rabbit-anti-DTor antibody (4% paraformaldehyde in 0.1 M PB, 1 : 200), mouse-anti-Synapsin (Developmental Hybridoma Study Bank (DHSB), University of Iowa, Iowa city, IA, USA; 4% paraformaldehyde in 0.1 M PB, 1 : 20), and mouse anti-DFMRP (DSHB, 4% paraformaldehyde in 0.1 M PB, 1 : 50). In addition, donkey anti-rabbit Alexa 568 and anti-mouse Alexa 488 were used as secondary antibodies at a dilution of 1 : 200 (Thermo Fisher Scientific, Waltham, MA, USA). Confocal images were taken with an LSM 700 (Zeiss, Oberkochen, Germany) and processed with an LSM image browser (Zeiss) and Adobe Photoshop (Adobe Systems Inc., San Jose, CA, USA).

### 2.4. Western Blot Analysis

Adult heads and larval body wall muscle preparations with brains were ground in RIPA buffer (150 mM NaCl, 1% IGEPAL CA-630, 0.5% Na-deoxycholate, 0.1% SDS, 50 mM Tris, pH 8.0; Sigma-Aldrich) with protease inhibitor cocktail (Roche Diagnostics, Mannheim, Germany). Twenty micrograms of protein was separated using a 12% SDS-PAGE gel and transferred onto a nitrocellulose membrane. Rabbit-anti-DTor antibodies (1 : 5,000), mouse anti-DFMRP (1 : 200, DSHB), mouse anti-*α*-Tubulin antibodies (1 : 5,000, DSHB), and rabbit anti-Alcohol dehydrogenase (ADH) antibodies (1 : 500, Santa Cruz Biotechnology, Dallas, Texas, USA) were used. The intensities of all bands were semiautomatically measured using the wander tools and histogram functions in Adobe Photoshop (Adobe Systems Inc.).

### 2.5. PNGase F Treatment

Proteins from larval body wall muscle preparations were extracted and then treated with PNGase F according to the manufacturer's protocol (New England Biolabs Inc., Ipswich, MA, USA). Briefly, 100 *μ*g of protein was mixed with glycogen denaturing buffer (New England Biolabs Inc.) and incubated for 10 min at 94°C. After adding NP-40, G7 buffer, BSA, and PNGase F (New England Biolabs Inc.), samples were incubated for 4 hr at 37°C. After boiling with SDS sample buffer, samples were loaded on a 12% SDS-PAGE gel. Rabbit anti-DTor antibodies were used to detect DTor proteins.

### 2.6. Behavioral Assays

One group of flies consisting of five males and five females of identical ages was tested for locomotor abilities as described in the literature [5]. After flies were transferred into a vial, the vials were transferred into a preheated 38°C water bath. The locomotor ability of each fly was determined, that is, whether it was climbing, walking, or staying at the bottom in vial at each time point. In total 120 flies were collected from three independent crosses and were raised at 30°C to accelerate the aging process. Gal4 driver flies were crossed with* w1118*,* UAS-DTor-cDNA*, or* pWiz-DTor-RNAi* flies and then compared. Two-sample *t*-tests performed using Minitab software (Minitab Inc.) were employed to determine differences in locomotor ability among experimental and control flies.

### 2.7. Oxidative Stress Test

One hundred to 120 female flies were collected from three independent crosses. The following genotypes were examined:* Tub-Gal4/+, UAS-DTor-cDNA/+; Tub-Gal4/Tb, Tub-Gal4/pWIZ-DTor-RNAi*, and* Tub-Gal4/UAS-HTor1A* ^*WT*^. Adult flies were tested on a sucrose food (1% agar, 5% sucrose, DaeJung Chemicals & Metals Co. Ltd.) containing 1% H_2_O_2_ (Merck, Whitehouse Station, NJ, USA) or 10 mM paraquat (Sigma-Aldrich, St. Louis, MO, USA). The number of live flies was counted every 24 hours. Kaplan-Meier survival analysis was performed to obtain hazardous ratios and *p* values using R (http://www.r-project.org/) as previously described [13].

### 2.8. 2-Dimensional Electrophoresis and Proteomic Analysis

Proteins are differentially expressed between* Tub-Gal/+* and* pWIZ-DTor-RNAi/+*;* Tub-Gal4/+* were examined by 2-dimensional (2D) electrophoresis. Protein extracts were obtained from 10-day-old adult fly heads by homogenizing for 5 min using a manual pestle in 100 *μ*L lysis buffer [8.0 M urea, 18 mM DTT, 4% (w/v) CHAPS, 40 mM Tris-HCl (pH 8.0), 10 mM EDTA, 0.5% IPG buffer (pH 4~7, GE Healthcare, Buckinghamshire, England)] with protease inhibitor cocktail (Roche Diagnostics, Basel, Switzerland). Then, 300 *μ*L lysis buffer was added and supernatant was harvested by spinning down at 15,000 ×g for 10 min at 4°C. After the protein concentration was quantified using the Bradford assay, 200 *μ*g protein was loaded on immobilized linear gradient strips (pH 4–7) using the IPGphor system (GE Healthcare). After the strips were rehydrated for 12 hr, focusing was accomplished in three steps: 200 V for 1 hr, 500 V for 1 hr, and 8,000 V for 8 hr. Before running the 2nd dimension on a 12% poly-acrylamide SDS gel using an Ettan DALT electrophoresis kit (GE Healthcare), strips were incubated in 1.5 M Tris-HCl buffer (pH 8.8, 10% SDS, 87% glycerol, 6 M urea, and 64.8 mM DTT) for 15 min with gentle shaking. Two D gels were stained with 0.1% Coomassie Brilliant Blue 250 and then scanned with a PowerLook III image scanner (UMAX Data Systems, Hsinchu, Taiwan). Scanned images were analyzed using Progenesis Editor software (Nonlinear Dynamics Ltd., Newcastle, UK) with the exclusion filter set manually. The total numbers of protein spots in 6 gels ranged within 849~1076 (*Tub-Gal4/+* No. 1, 1076; No. 2, 849; No. 3, 1060;* pWIZ-DTor-RNAi/+; Tub-Gal4/+* No. 1, 902; No. 2, 982; No. 3, 1071). The spots for subsequent protein identification analysis were selected by comparing normalized volumes of protein spots from three different comparisons using a one-way analysis of variance (ANOVA; threshold of significance, *p* < 0.05).

Six differentially expressed spots were excised and washed with 10 mM NH_4_HCO_3_ and 50% CH_3_CN (Sigma-Aldrich), and then they were digested with 12.5 ng/mL Trypsin Gold (Promega, Madison, WI, USA) in buffer (50 mM NH_4_HCO_3_, 5 mM CaCl_2_) at 37°C for 12~15 hr. Digested peptides were recovered by extracting twice with 50 mM NH_4_HCO_3_ and 100% CH_3_CN. Extracted solutions were pooled, lyophilized, and stored at 4°C.

The proteins presented in spots were identified by MS/MS experiments performed with a nano-LC/MS system composed of a Surveyor HPLC system (Thermo Fisher Scientific) and an electrospray ionization- (ESI-) quadrupole ion trap (IT) mass spectrometer (MS) (LCQ Deca XP-Plus, ThermoFisher Scientific) equipped with a nano-ESI source. A C18 trap column (ID 300 *μ*m, length 5 mm, particle size 5 *μ*m; DIONEX/LC Packings, Sunnyvale, CA) was used for desalting and concentrating 10 *μ*L digested peptide samples at a flow rate of 20 *μ*L/min. Trapped peptides were separated by back-flushing into a home-made microcapillary column (150 mm in length) packed with C18 resin (particle size 5 *μ*m) in 75 mm silica tubing (8 mm ID orifice) using mobile phase A (0% CH_3_CN, 0.02% HCO_2_H, 0.5% CH_3_COOH) and B (80% CH_3_CN, 0.02% HCO_2_H, and 0.5% CH_3_COOH). The gradient elution was started with 5% of the mobile phase B and 95% of the mobile phase A for 15 min. Mobile phase B was increased to 20% for 3 min, 50% for 32 min, 60% for 5 min, 80% for 5 min, and 100% for 10 min. When samples were changed, the column was equilibrated and cleaned with 5% CH_3_CN for 10 min. The operating conditions for obtaining the MS and MS/MS spectra was a capillary temperature of 220°C, an ESI voltage of 2.5 kV, and a collision energy setting of 35%. The three most abundant MS ions were selected as peaks and became the targets of MS/MS analysis with 180-second dynamic exclusion. MS scan functions and HPLC solvent gradients were controlled by an Xcalibur data system (Thermo Fisher Scientific). A* Drosophila* nonredundant protein database at NCBI (ftp://ftp.ncbi.nlm.nih.gov/, May 2009 Released version, the number of entries were 220,666) was used for searching MS/MS spectra using SEQUEST (version 3.3.1, Thermo Fisher Scientific) with search algorithm from the Bioworks software package (version 3.3.1, Thermo Fisher Scientific). Search parameters were the following: a mass tolerance for parental ions was 2.0 Da or the MS/MS was 1.0 Da, one missed cleavage per peptide was allowed, and protein modifications were not considered. Matched peptide sequences were deduced based on the b and y ions and filtered using the cross-correlation score (*X*corr) and the normalized difference in cross-correlation scores (ΔCn) based on following thresholds: (1) the unique ΔCn > 0.1, (2) minimum *X*corr of 1.9, 2.2, and 3.75 for charge states +1, +2, and +3, respectively. Proteins were only considered to be identified if they had at least two matched peptides.

### 2.9. Coimmunoprecipitation

To examine whether Torsin and FMRP were present in the same protein complexes, rabbit anti-DTor antibodies, rabbit anti-HTor1A antibodies [5, 19], mouse anti-DFMRP antibodies, and mouse anti-FMR1 antibodies were used for coimmunoprecipitation. Protein extracts from adult heads or larval body wall muscle preparations with brains were precleared with normal rabbit or mouse IgG agarose beads (Santa Cruz Biotech., Santa Cruz, CA, USA) and protein A/G plus agarose beads (Santa Cruz Biotech.). After mixing with 20 *μ*L mouse anti-DFMRP antibodies at 4°C overnight, protein A/G agarose beads preblocked with 1% bovine serum albumin (Amresco Inc., Solon, OH, USA) in RIPA buffer were added. We also performed control experiments without primary antibodies. After incubation at 4°C for 2 hr with shaking, agarose beads were precipitated by centrifuging them at 15,000 rpm for 10 minutes. After the beads were washed 3 times with RIPA buffer, sample buffers were added, and the samples were boiled before being loaded on a 12% SDS-PAGE gel. Proteins were transferred onto a nitrocellulose membrane and then probed with appropriate antibodies.

Two independent human plasma samples purchased from Innovative Research (Novi, MI, USA) were used for HTor1A and FMR1 coimmunoprecipitation experiments. Ten microliter of rabbit anti-HTor1A antibodies or mouse anti-FMR1 antibodies was added to 50 *μ*L of precleaned human plasma as described above. After mixing with protein A/G agarose beads for 2 hr at 4°C, beads were precipitated by centrifuging at 13,000 ×g for 10 min. We also performed control experiments without primary antibodies. After being washed 3 times with RIPA buffer, beads were boiled with SDS sample buffer and loaded on 12% SDS-PAGE gels. Proteins were transferred onto a nitrocellulose membrane and then probed with antibodies. The heavy chains of mouse IgG or rabbit IgG-specific HRP conjugated secondary antibodies (Thermo Fisher Scientific) were used to avoid detection of the IgG light chains.

Control experiments without primary antibodies did not show any DTor, DFRMP, FMR1, or HTor1A bands (Supplementary Figure 2).

## 3. Results

### 3.1. Two Different Isoforms of* Drosophila* Torsin Proteins in* Drosophila*

**Figure 1:**
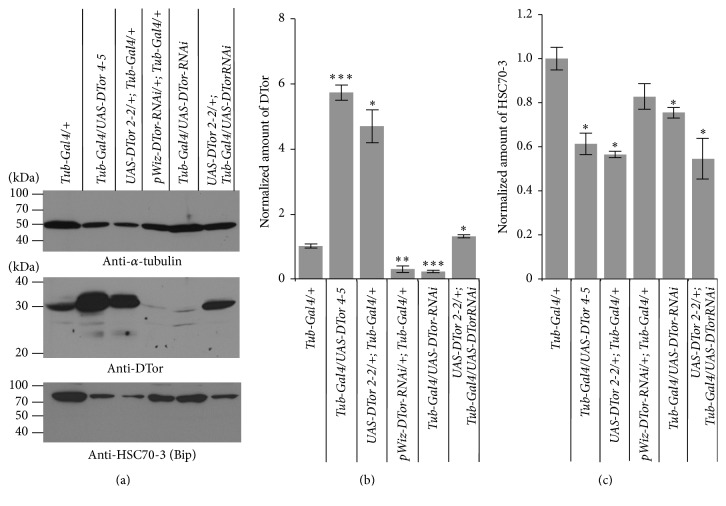
The altered expression levels of* Drosophila* Torsin protein in transgenic flies. (a) The western blots of various strains expressing* UAS-DTor-cDNA, pWiz-* or* UAS-DTor-RNAi*, or both of* UAS-DTor-cDNA* and* UAS-DTor-RNAi* in all tissues using Tub-Gal4 drivers. UAS-DTor lines increased expression of DTor compared to that of genetic background (Tub-Gal4/+). Expressing two independent DTor-RNAi lines using Tub-Gal4 drivers induced significantly reduced DTor. At least 2 different sizes of DTor bands were detected from genetic backgrounds and flies expressing DTor-RNAi. However, 1 or 2 additional bands were detected from UAS-DTor overexpressing lines. The expression of the* Drosophila* homologue of Bip and heat shock 70-kDa cognate protein 70-3 (HSC70-3) was reduced in most transgenic flies compared with that of controls. (b) Quantification of the normalized amount of DTor in various strains. Two* UAS-DTor-cDNA* lines had significantly increased expression levels compared to those of Tub-Gal4/+. In contrast, the expression of DTor proteins was downregulated when two independent double-stranded RNA inhibitory constructs for DTor were expressed by Tub-Gal4 promoters. Simultaneous expression of both DTor-cDNA and DTor-RNAi constructs produced DTor expression levels approximately 1.5 times higher than those of genetic back ground (Tub-Gal4/+). (c) The expression of HSC70-3 (Bip) was significantly reduced in all transgenic fly, except one expressing* pWIZ-DTor-RNAi*. ^*∗*^ *p* < 0.05, ^*∗∗*^ *p* < 0.01, and ^*∗∗∗*^ *p* < 0.005.

The expected molecular weight of* Drosophila* Torsin (DTor) proteins based on the deduced amino acid sequence is 38.17 kDa. The size of endogenous DTor protein detected by western blot analysis was approximately 32~35 kDa (Figure 1(a)). To investigate the* in vivo* function of DTor, two independent* UAS-DTor-cDNA* lines were generated and crossed with* Tub-Gal4* driver flies to produce flies that ubiquitously expressed DTor proteins. The two* UAS-DTor-cDNA* lines had significantly increased DTor protein levels compared to those of control genetic background flies (*Tub-Gal4/+*; Figures 1(a) and 1(b)). In addition, expression of two independent DTor-specific double-stranded inhibitory RNA (DTor-RNAi) constructs driven by Tub-Gal4 promoters significantly reduced expression of endogenous DTor to less than 30% of DTor proteins that were detected in the controls. Combined expression of* UAS-DTor-cDNA* and* DTor-RNAi* constructs caused DTor expression to be 1.5 times higher than the expression seen in flies of control genetic background.

**Figure 2:**
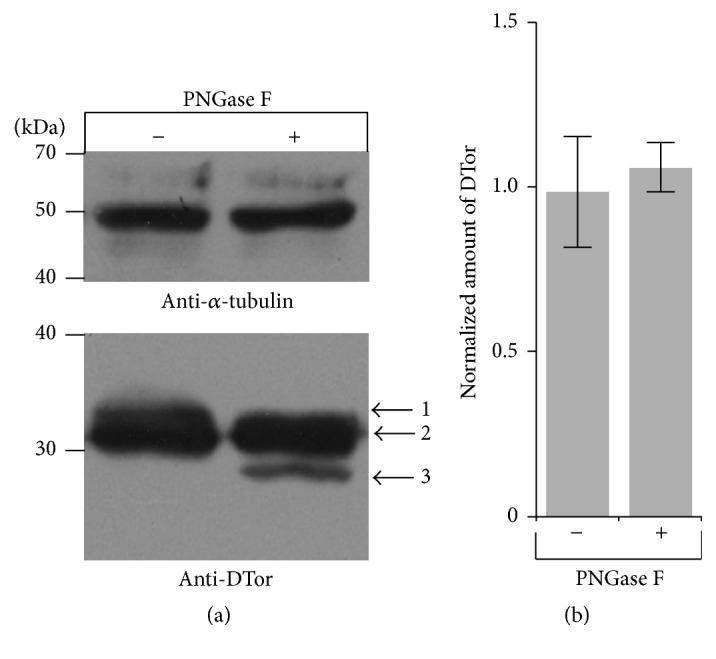
DTor is an N-glycosylated protein. (a) When protein extracts from* w1118* were treated with PNGase F, the upper band indicated by arrow 1 disappeared and the lower band indicated by arrow 3 appeared. (b) The PNGase F treatment did not alter the overall amount of DTor protein.

The cDNA of DTor is known to consist of two exons (http://www.flybase.org/). There are several methionine (Met) residues at the beginning of DTor suggesting that the Met at 2nd, 6th, or 125th positions may be used to initiate translation of DTor protein, which may contribute to the several different sizes of DTor bands observed. Another possible cause of several sizes of DTor proteins is glycosylation. Thus, we examined the possible glycosylation of DTor using N-, O-, and C-glycosylation prediction servers [20, 21]. Even though two potential N-glycosylation sites (Asparagine (Asn) positions at 96 and 311) were present in DTor, only the Asn at 96 was predicted to be N-glycosylated (Supplementary Figure 1). To confirm whether some DTor proteins in* Drosophila* were N-glycosylated, protein extracts were treated with PNGase F before performing anti-DTor western blot analysis. Band 1 appeared in nontreated samples, and Band 3 was clearly present in treated samples (Figure 2). However, band 2 was unchanged by treatment. These results suggested that DTor may be modified by N-linked glycosylation and other posttranslational modifications of DTor proteins, which are not yet known.

### 3.2. The Synaptic Morphology Was Altered in Flies with Different Levels of DTor Proteins

**Figure 3:**
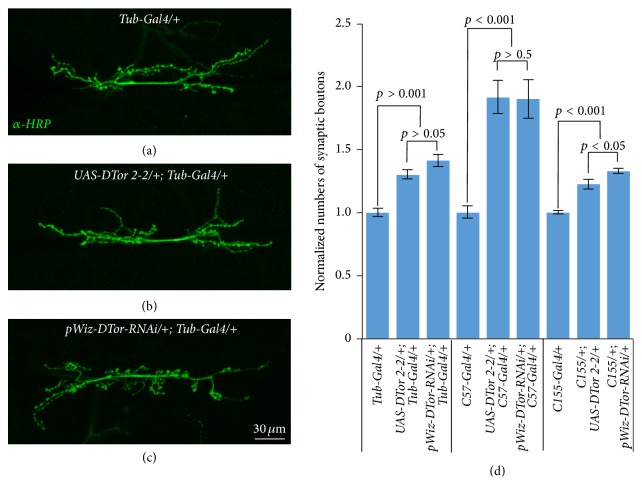
Altered NMJ structures in DTor mutants. (a–c) Representative NMJ morphologies from (a) control (*Tub-Gal4/+*), (b) DTor-cDNA overexpression (*UAS-DTor 2-2/+; Tub-Gal4/+*), and (c) DTor-RNAi expression (*pWiz-DTor-RNAi/+; Tub-Gal4/+*) NMJs. (d) Quantification of the number of synaptic boutons when DTor-cDNA or DTor-RNAi were expressed ubiquitously (Tub-Gal4); postsynaptically, in the muscle (C57-Gal4); and presynaptically, pan-neuronally (C155-Gal4). Alteration of DTor proteins at pre- and/or postsynaptic terminals significantly increased the number of synaptic boutons.

To examine any consequence of altered expression levels of DTor in the neuronal system, we examined the number of synaptic boutons in Type 1 glutamatergic larval NMJs. Ubiquitous expression of either DTor-cDNA or DTor–RNAi by Tub-Gal4 drivers significantly increased the numbers of synaptic boutons compared with those of control NMJs (*Tub/+, *99.7 ± 2.72;* UAS*-*DTor-cDNA/+*;* Tub/+*, 129.6 ± 3.5, *p* < 0.001;* pWiz-DTor-RNAi/+*;* Tub/+*, 140.6 ± 4.73, *p* < 0.001) (Figures 3(a)–3(d)). The postsynaptic terminal-specific expression of DTor-cDNA or DTor–RNAi by C57-Gal4 drivers significantly increased synaptic bouton numbers to a greater degree (*C57/+, *75.62 ± 3.25;* UAS*-*DTor-cDNA/+; C57/+, *144.63 ± 9.67, *p* < 0.001;* pWiz-DTor-RNAi/+; C57/+*, 140.6 ± 4.73, *p* < 0.001; Figure 3(d)). Pan-neuronal expression of DTor-cDNA or DTor–RNAi by* C155-Gal4* drivers also significantly increased synaptic bouton numbers (*C155/+*, 84 ± 1.06;* C155/+; UAS*-*DTor-cDNA/+*, 102.4 ± 3.06, *p* < 0.001; C155/+;* pWiz-DTor-RNAi*/+, 111.25 ± 1.56, *p* < 0.001). These results suggested that maintenance of DTor protein levels within normal ranges might be important for proper formation of synaptic structures.

### 3.3. Reduced Expression of DTor Protein Was Involved in Locomotor Control

**Figure 4:**
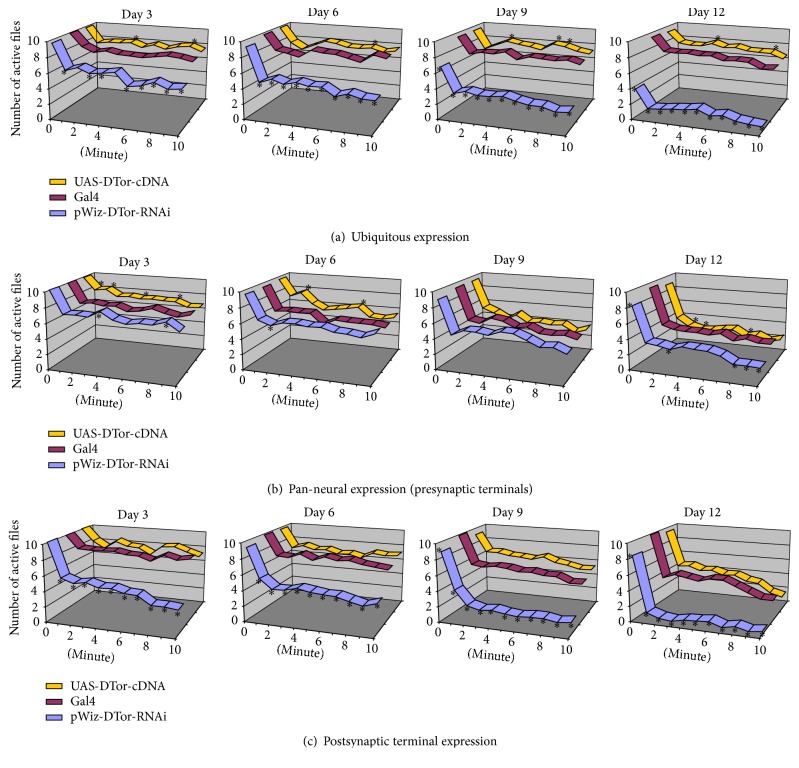
Reduced expression of DTor impaired locomotor behavior. Flies of different ages expressing DTor-cDNA or DTor-RNAi under control of various Gal4 drivers were monitored in vials at 38°C for 10 min. The number of active flies was plotted at each time point. Asterisks indicate the time points showing a significant difference (*p* < 0.05) from *t*-test analysis. (a) Tub-Gal4 drivers, (b) C155-Gal4 drivers, and (c) C57-Gal4 drivers. Ten to twelve groups of* Tub-Gal4/+, UAS-DTor-cDNA/+; Tub-Gal4/+, pWIZ-DTor-RNAi/+; Tub-Gal4/, C155-Gal4, C155-Gal4; UAS-DTor-cDNA/+, C155-Gal4; pWIZ-DTor-RNAi/+, C57-Gal4/+, UAS-DTor-cDNA/+; C57-Gal4/+,* and* pWIZ-DTor-RNAi/+; C57-Gal4/+* were collected from three independent crosses and examined.

Next, we tested whether altered expression of DTor in* Drosophila* might cause any locomotor defect. When DTor-RNAi constructs were expressed ubiquitously using Tub-Gal4 drivers, flies showed early death compared with control flies or DTor-cDNA expressing flies. From 9 days of age, significantly fewer flies (6.50 ± 0.529) survived compared with genetic control flies (9.417 ± 0.229) or DTor-cDNA expressing flies (9.417 ± 0.229; Figure 4(a)). The loss of motor control in DTor-RNAi expressing flies manifested at 3 days of age. Compared with control or DTor-cDNA expressing flies, significantly fewer flies maintained their motor control (Figure 4(a)). Those motor-defects grew more severe as the flies aged. Bay day 12, 2 or fewer flies maintained their motor control even though at least 8 control or DTor-cDNA expressing flies still retained their motor control (Figure 4(a)). We further characterized whether loss of motor control in DTor-RNAi expressing flies was caused by reduced expression of DTor proteins in the nervous system or in the muscles (Figures 4(b) and 4(c)). When DTor-RNAi was expressed in the nervous system, the significant differences in the numbers of live flies were not observed until Day 12 (C155/+; DTor-RNAi/+, 8.0 ± 0.43) compared with control flies (C155/+, 9.364 ± 0.20). However, loss of motor control was not always observed. On Day 12, loss of motor control was observed at only a few time points for DTor-RNAi or DTor-cDNA expressing flies (Figure 4(b)).

Similarly, when DTor-cDNA or DTor-RNA was expressed in the muscles, early death of flies was not observed, although more flies expressing DTor-RNAi in all muscle (8.750 ± 0.392) died compared with control flies (*C57/+*, 9.498 ± 0.133), but not compared with DTor-cDNA expressing flies (*UAS-DTor-cDNA/+; C57/+*, 8.833 ± 0.322), from Day 9. Interestingly, the loss of motor control in DTor-RNAi-expressing flies is manifested by Day 3 (Figure 4(c)). These behavioral analysis data suggested that the early death of flies required the loss of DTor protein from both the neurons and muscles, but reduction of muscular expression of DTor proteins may contribute to the loss of motor control.

### 3.4. Resistance to Chemical Stressors Was Changed in Flies with Altered Expression Levels of Torsin Proteins

**Figure 5:**
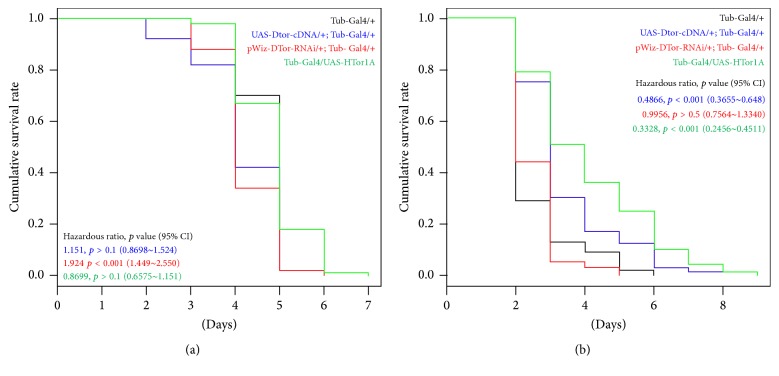
Kaplan-Meier survival analysis of chemically induced oxidative stress with different expression levels of DTor in* Drosophila*. (a) When flies were treated with H_2_O_2_, only DTor-RNAi expressing flies showed significantly increased HR. (b) When flies were treated with paraquat, DTor-cDNA, or HTor1A-cDNA-expressing flies showed significantly reduced HR.

To understand the physiological consequences of altered expression levels of Torsin, we treated flies with two oxidative stressors. When flies were exposed to H_2_O_2_, the hazardous ratio (HR) of flies ubiquitously expressing DTor-RNAi (Figure 5(a)) was 1.924 (95% confidence interval (CI), 1.449~2.550) compared with control flies. However, the HRs of other flies were not significantly different from those of control flies.

Flies expressing DTor-cDNA or HTor1A-cDNA showed increased resistance to paraquat. The HRs of flies expressing DTor- or HTor1A-cDNA were 0.4866 (95% CI, 0.3655~0.6480) and 0.3328 (95% CI, 0.2456~0.4511), respectively, compared with control flies (Figure 5(b)). However, flies expressing DTor-RNAi were not significantly different (HR, 0.9956; 95% CI, 0.7564~1.3340) from control flies. These chemical stressor experiments suggested that an increased total amount of Torsin proteins might contribute to increase resistance to paraquat, but not H_2_O_2_.

### 3.5. Altered Expression of ADH in DTor Mutant Flies

**Figure 6:**
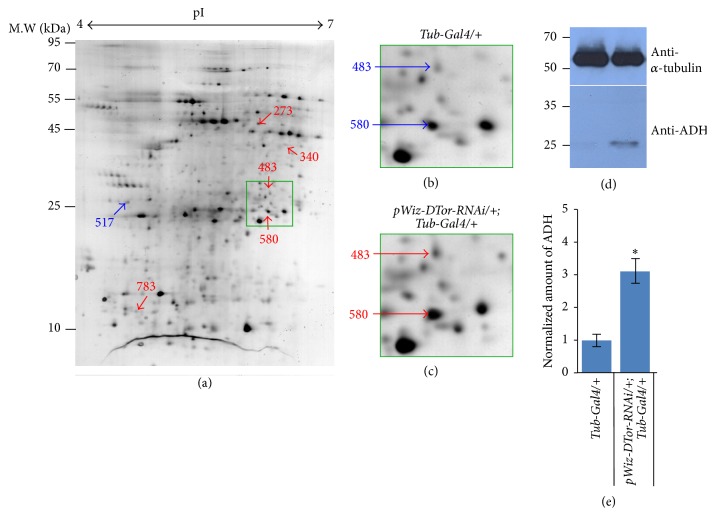
2D analysis of proteins extracted from* Drosophila* adult heads. (a) Representative 2D electrophoresis image of protein extracts from control adult heads. A total of 6 spots were dysregulated in DTor-RNAi expressing fly brains. Blue arrows indicated spots with decreased volumes in DTor-RNAi expressing flies compared with controls. Red arrows point to spots with increased volumes in DTor-RNAi-expressing flies compared with controls. (b) Spots 483 and 580 in a control gel. (c) Spots 483 and 580 in a DTor-RNAi gel showing a comparatively larger volume. (d) Anti-ADH western blot analysis of genetic back ground and DTor-RNAi-expressing flies. (e) ADH protein levels detected from protein extracts from DTor-RNAi-expressing flies were 3.1-fold of those of control fly brains. ^**∗**^ *p* <0.05.

**Figure 7:**
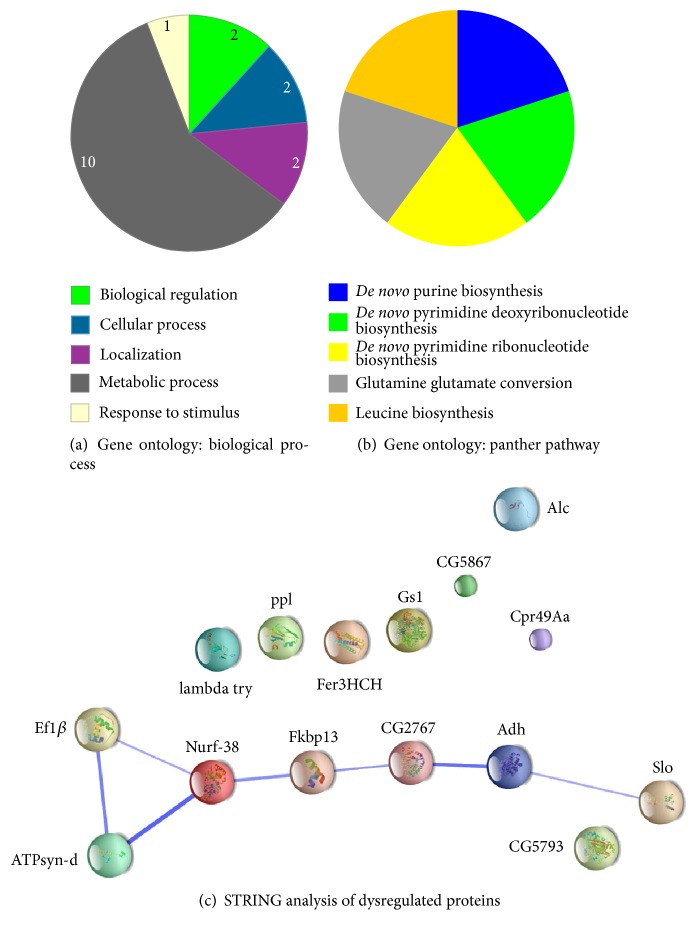
Gene ontology and interacting gene analysis for 22 dysregulated proteins in DTor mutant brains. (a-b) Twenty-two dysregulated proteins were allocated according to their biological processes (a) or pathways (b). Protein-protein interactions among them were predicted using STRING DB (c). The size of each node is correlated with the amount of structural information associated with each protein. The width of lines represents the confidence score of precalculated protein-protein interaction [16, 17].

To gain further insight into the role of DTor in fly neuronal systems, unbiased 2D proteomic approaches were employed. A total of 6 spots from DTor-RNAi gels had significantly different volumes compared with those from control gels (Figures 6(a)–6(c)). The list of proteins identified from dysregulated spots was summarized in Table 1 and categorized using the PANTHER Classification System (http://www.pantherdb.org/) [22] according to biological functions and pathways (Figures 7(a)-7(b)). Of the 15 dysregulated proteins, 10 proteins were allocated into metabolic processes. Two proteins were allocated into biological regulation, cellular processes, or localization. One protein was related to response to stimuli. Signaling pathways that those dysregulated proteins were categorized into were* de novo* purine biosynthesis,* de novo* pyrimidine deoxyribonucleotide biosynthesis,* de novo* pyrimidine ribonucleotide biosynthesis, glutamine glutamate conversion, and leucine biosynthesis. In addition, the protein-protein interactions among 15 dysregulated proteins were examined using the Search Tool for the Retrieval of Interacting Genes (STRING; http://www.string-db.org/) database [16]. Slo, ADH, CG2767, Fkbp13, Nurf-38, ATPsyn-d, Ef1*β*, and HSC70-4 showed strong association among those dysregulated proteins in DTor mutant brains. Because ADH was detected from several spots and is also known to function in metabolism, we further investigated by performing anti-ADH western blot analysis. Consistent with the 2D proteomic results, the normalized amount of ADH in DTor-RNAi expressing brains was increased 3.1-fold (3.11 ± 0.38, *p* < 0.05; Figures 6(d) and 6(e)). These data suggested that reduced DTor significantly altered metabolism in adult* Drosophila* brains.

### 3.6. DTor Proteins Formed Protein Complexes with Fragile-X-Mental Retardation Proteins

**Figure 8:**
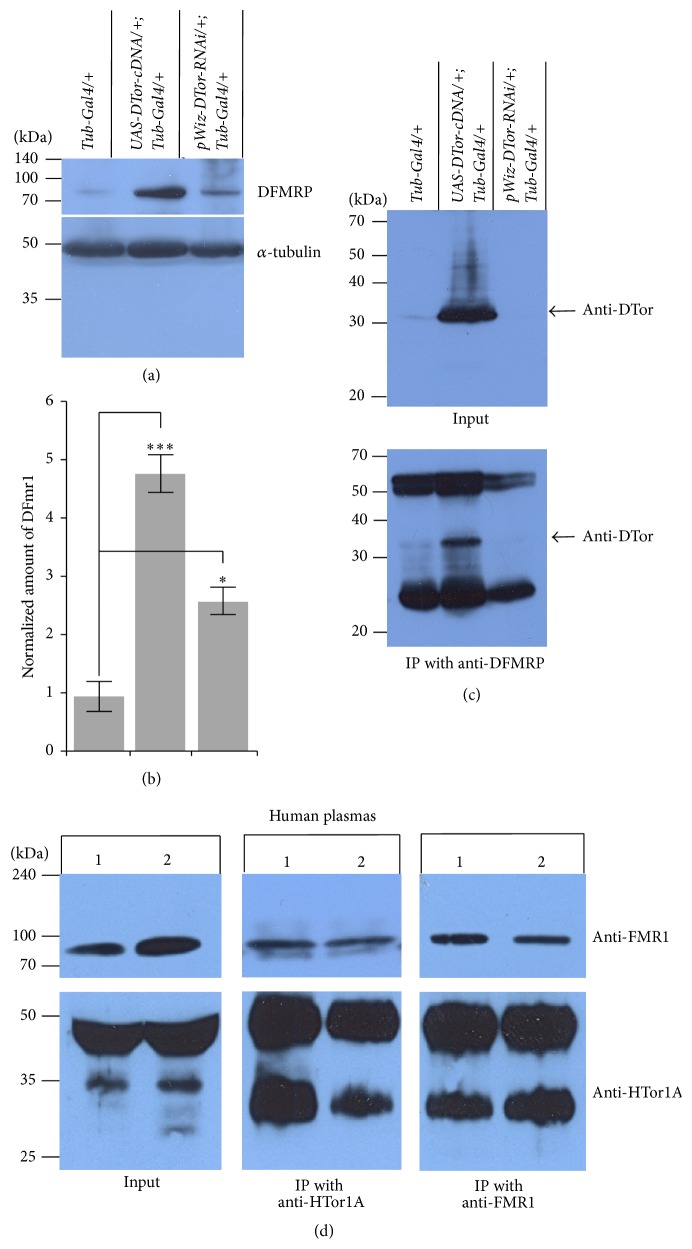
Torsin proteins existed in the same protein complexes as Fragile-mental retardation proteins in* Drosophila* brains and human blood plasma. (a) The expression of DFMRP proteins was altered in DTor-cDNA or DTor-RNAi-expressing brains. (b) Quantification of DFMRP proteins in control and DTor-cDNA or DTor-RNAi-expressing brains. (c) DTor and DFMRP proteins exist in the same protein complexes. (d) Human FMR1 and HTor1A proteins are present in the same protein complexes. ^**∗**^ *p* <0.05. ^**∗∗∗**^ *p* <0.005.

Because the expression of ADH was reduced in* dfmrp* mutants [23], we further examined whether expression of DFMRP in DTor-cDNA or DTor–RNAi expressing brains was altered. There was 5.1-fold or 2.7-fold increase in the normalized amount of DFMRP in DTor-cDNA or DTor–RNAi expressing brains, respectively, compared with control brains (Figures 8(a) and 8(b)). Because this result raised the possibility of an interaction between DTor and DFMRP, we performed coimmunoprecipitation (Co-IP) experiments to examine whether DTor and DFMRP existed in the same protein complex. When mouse anti-DFMRP antibodies were used for IP, a strong DTor band was detected from DTor-cDNA expressing brains. A weak but definite band was detected from the control brains. However, a DTor band was not detected from DTor-RNAi expressing brains (Figure 8(c)). In addition, without primary antibodies, DTor bands were not detected (Supplementary Figure 2(A)).

We further tested the interaction between HTor1A with FMR1 by performing Co-IP experiments using human plasma. Strong HTor1A and FMR1 bands were detected from two independent human plasma samples. When anti-HTor1A antibodies were used for IP, strong FMR1 bands were detected. In addition, when mouse anti-FMR1 antibodies were used for IP, strong anti-HTor1A bands were detected. Without primary antibodies, no HTor1A or FMR1 bands were detected (Supplementary Figure 2(B)). These results suggested that coexistence of Torsin proteins and FMR1 proteins in the same protein complex might be an evolutionarily conserved phenomenon.

### 3.7. The NMJ Phenotype of DTor-RNAi Expressing* dfmrp* Mutants Was Similar to That of* dfmrp* Mutants

**Figure 9:**
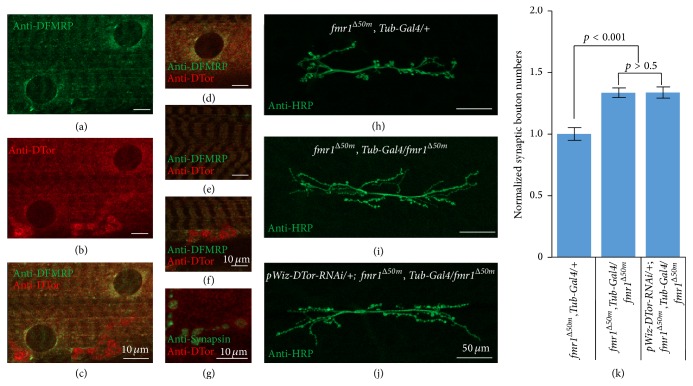
Colocalization and genetic interaction between DTor and DFMRP in* Drosophila*. (a–c) Localization patterns of DTor (red) and DFMRP (green) in* w1118* larval muscles. DTor and DFMRP proteins are partially colocalized in muscle organelles. They are partially colocalized at (d) the outside of nuclear membranes in muscles and (e) muscle fibers. However, they are not colocalized at synaptic boutons (f). (g) A small portion of DTor protein at the synaptic boutons was colocalized with Synapsin. (h–j) Representative NMJ morphology of genetic background larvae (*fmr1* ^Δ*50m*^,* Tub-Gal4*/+),* dfmrp* mutant larvae (*fmr1* ^Δ*50m*^,* Tub-Gal4*/*fmr1* ^Δ*50m*^), and* dfmrp* mutant larvae expressing DTor-RNAi (*pWiz-DTor-RNAi/+; fmr1* ^Δ*50m*^ *Tub-Gal4*/*fmr1* ^Δ*50m*^). (k) The quantification of the normalized number of synaptic boutons from three mutant NMJs.

The presence of DTor and DFMRP in the same protein complexes raised the possibility that DTor and DFMRP might act via the same signal transduction pathway regulating synaptic plasticity. First, we examined whether DTor and DFMRP localized to the same cellular compartments by performing double-labeling immunohistochemistry using anti-DTor and anti-DFMRP antibodies (Figures 9(a)–9(f)). Most of the DTor and DFMRP immunoreactivity was colocalized throughout muscles (Figures 9(a)–9(c)) with strong signals outside of nuclear membranes (Figures 9(a)–9(d)) or muscle fibers (Figure 9(e)). Even though strong DTor signals (red) were detected from synaptic boutons, DFMRP signals (green) did not colocalize (Figure 9(f)). Double-labeling with mouse anti-Synapsin antibodies revealed that only a small portion of DTor was colocalized with Synapsin at the presynaptic terminals. The colocalization of DTor and DFMRP in muscle nuclei and fibers suggested that they might regulate synaptic plasticity by controlling signaling from NMJs to nuclei. Therefore, we examined synaptic bouton numbers in DTor-RNAi expressing* dfmrp mutant* NMJs (*pWiz-DTor-RNAi/+; fmr1* ^Δ*50m*^,* Tub-Gal4*/*fmr1* ^Δ*50m*^) and compared them with* dfmrp* mutant (*fmr1* ^Δ*50m*^,* Tub-Gal4*/*fmr1* ^Δ*50m*^) or control genetic background (*fmr1* ^Δ*50m*^,* Tub-Gal4*/+) NMJs (Figures 9(h)–9(j)). We found that expression of DTor-RNAi in* dfmrp* mutant did not produce a synergistic phenotype. The numbers of synaptic boutons in double mutant NMJs (129.7 ± 4.3) were similar to those of* dfmrp* mutant NMJs (129.6 ± 3.6, *p* value > 0.5; Figure 9(k)). Taken together, DTor and DFMRP might be present in the same signaling pathway regulating NMJ development and extensions.

## 4. Discussion

The major goal of this investigation was to elucidate the* in vivo* function of Torsin family proteins in the regulation of locomotor ability and structural synaptic plasticity. The most significant finding in this study was that the downregulation of DTor expression was sufficient to increase the fly's susceptibility to environmental stress, which evoked locomotor disabilities. However, our finding that reduced expression of DTor in all tissues or specifically in muscles, but not specifically in neurons, was sufficient to cause increased susceptibility to environmental stress (Figure 4) was contrary to our expectation, because it has been shown that the expression of mutant HTor1A in* Drosophila* increased the fly's susceptibility [5, 13, 19]. DTor proteins are abundantly expressed in larval and adult brains [24], and the DTor null mutant was semilethal in flies, with the few surviving homozygous adults being sterile and exhibiting locomotor defects [25]. How can we solve discrepancy between our data and previous results? One possible interpretation could be that fly neurons that regulate motor controls might have resistance to the reduced amount of DTor. This interpretation might also explain why only 30~40% of ΔE HTor1A heterozygote mutant carriers in humans manifested the severe behavioral symptoms. Similar to fly neurons showing resistance to lower level of DTor, neurons in human brains involved with DYT1 dystonia might be resistant to a reduced level of normal HTor1A. Thus, 50% of normal HTor1A could be sufficient to maintain proper motor control in humans unless another mutation that could trigger manifestation of DYT1 dystonia is present.

Another important finding in this study is the biochemical and genetic interaction between DTor and DFMRP in fly brains and NMJs (Figures 8 and 9). Interestingly, DTor and DFMRP were present in the same protein complexes, and their colocalization in various cellular organelles such as muscle nuclear membrane and muscle fibers is very striking. In addition, the phenotype of* dfmrp* null mutants expressing DTor-RNAi was similar to those of* dfmrp* null mutant or DTor-RNAi NMJs, suggesting that DTor and DFMRP act through the same signal transduction pathway regulating synaptic plasticity. Even though we confirmed the biochemical interaction between HTor1A and FMR1 in human plasma in this study (Figure 8), the possible genetic interaction between these two proteins in humans could be supported by the fact that the age of onset for DTY1 dystonia is in adolescence. FMRP family proteins are known to play pivotal roles in nucleocytoplasmic trafficking of mRNA, dendritic localization of mRNA, and negative translational regulation [26]. In contrast to Fragile-X mental retardation, which is mainly caused by reduced expression of FMRP in human brains [26], the amount of FMR1 in DYT1 dystonia patients might be increased and results in reduced translation of mRNAs that might be important for proper development and maintenance of neural circuits and synaptic architecture. Thus, it will be intriguing to test this hypothesis in mouse transgenic models or human patients in the near future.

Unknown signal transduction pathways or components are often revealed by unbiased proteomic profiling experiments [27]. In this study, we found that the amount of ADH was altered in DTor-RNAi expressing fly brains compared with those of control brains (Figure 6). ADH is an evolutionarily conserved enzyme responsible for breaking down toxic alcohols to generate various metabolites such as aldehydes and ketones with the reduction of nicotinamide adenine dinucleotide (NAD^+^) to NADH in humans and animals [28]. Thus, an increased amount of ADH in fly brains might increase the amount of aldehydes or ketones. Recent studies in human neurological disorders suggested that many neurological disorders accompany metabolic changes in brains. For example, significant metabolic changes were reported from human brains with Alzheimer's disease and other related neurodegenerative disorders [29], Parkinson's disease [30], motor neuron disorders [31], major depressive disorder [32], and so forth. Interestingly, increased glucose metabolism was reported from the putamen, anterior cingulate, and cerebellar hemispheres in DYT1 dystonia patients and carriers [33]. In addition, DTor null mutant flies had significantly reduced brain dopamine levels in and showed strong genetic interaction with GTP cyclohydrolase [25]. Even though we have not investigated whether other enzymes present in ADH-related metabolic pathway such as ALDH were increased in DTor mutant fly brains, recent studies of ALDH showed that it regulates a GABA synthesis pathway in human midbrain dopaminergic neurons that play key roles in locomotor control [34]. The key characteristic of DYT1 dystonia is excessive cocontraction of agonist and antagonist muscles during movements and impairment of inhibitory functions in several parts of patients brains has been reported [35]. Taken together, altered metabolism in DTor-RNAi-expressing fly brains could cause defects in inhibitory mechanisms in neuronal circuits that are responsible for motor controls.

## 5. Conclusion

In this study, we provide genetic, biochemical, molecular, and cellular evidence that DTor and FMRP are present in the same protein complexes and may act via the same signaling pathway regulating synaptic plasticity. In addition, by performing unbiased proteomic profiling and biochemical analyses, we found that the amount of ADH in DTor-RNAi-expressing fly brains was altered. Further studies using various* Drosophila* models for DYT1 dystonia will reveal unknown pathophysiological mechanisms underlying DTY1 dystonia that will provide new insight into this enigmatic disorder.

## Supplementary Material

Prediction of Glycosylation in DTor.

## Figures and Tables

**Table 1:** The summary of 2D proteomic analysis results.

Spot #	ANOVA (*p*)	Average normalized volumes (fold change; ≥1.5, ≤0.75)		Identified proteins	M.W. (kDa)/PI
		*Tub-Gal4/+*	*pWiz-DTor-RNAi/+; Tub-Gal4/+*		
580	0.006	1.261 × 10^4^	2.006 × 10^4^ (1.6)	CG5793	24.6/6.12
				Ferritin 1 heavy chain homologue	23.1/5.49
				ATP synthase, subunit d	20.2/6.10

783	0.012	4.806 × 10^4^	7.165 × 10^4^ (1.5)	Cuticular protein 49Aa	15.6/4.54
				lambda Try	29.6/4.67
				pumpless	18.0/4.94

273	0.014	5.604 × 10^4^	1.059 × 10^5^ (1.9)	Glutamine synthase 1	44.4/6.02
				Slowpoke	130.2/5.10

483	0.032	6.143 × 10^4^	1.226 × 10^5^ (2.0)	CG5867	29.6/5.63
				Alcohol dehydrogenase	27.7/8.18
				Alicorn	36.4/5.25

340	0.044	3.090 × 10^4^	5.049 × 10^4^ (1.6)	Nucleosome remodeling factor-38 kDa	37.9/6.57
				CG2767	39.1/5.80
				CG5867	29.6/5.63

517	0.046	2.026 × 10^5^	1.499 × 10^5^ (0.74)	Alcohol dehydrogenase	27.7/8.18
				FK506-binding protein 14 ortholog	23.9/4.54
				Elongation factor 1*β*	24.2/4.20

## References

[1] Fahn S., Bressman S. B., Marsden C. D., Marsden C. D., Fahn S. (1987). Classification and investigation of dystonia. *Movements Disorders 2*.

[2] Ozelius L. J., Hewett J. W., Page C. E. (1997). The early-onset torsion dystonia gene (*DYT1*) encodes an ATP-binding protein. *Nature Genetics*.

[3] Fahn S. (1991). The genetics of idiopathic torsion dystonia. *International Journal of Neurology*.

[4] Grundmann K., Reischmann B., Vanhoutte G. (2007). Overexpression of human wildtype torsinA and human DeltaGAG torsinA in a transgenic mouse model causes phenotypic abnormalities. *Neurobiology of Disease*.

[5] Koh Y.-H., Rehfeld K., Ganetzky B. (2004). A *Drosophila* model of early onset torsion dystonia suggests impairment in TGF-*β* signaling. *Human Molecular Genetics*.

[6] Goodchild R. E., Kim C. E., Dauer W. T. (2005). Loss of the dystonia-associated protein torsinA selectively disrupts the neuronal nuclear envelope. *Neuron*.

[7] Liang C.-C., Tanabe L. M., Jou S., Chi F., Dauer W. T. (2014). TorsinA hypofunction causes abnormal twisting movements and sensorimotor circuit neurodegeneration. *The Journal of Clinical Investigation*.

[8] Sharma N., Baxter M. G., Petravicz J. (2005). Impaired motor learning in mice expressing torsinA with the DYT1 dystonia mutation. *The Journal of Neuroscience*.

[9] Grundmann K., Glöckle N., Martella G. (2012). Generation of a novel rodent model for DYT1 dystonia. *Neurobiology of Disease*.

[10] Page M. E., Bao L., Andre P. (2010). Cell-autonomous alteration of dopaminergic transmission by wild type and mutant (ΔE) TorsinA in transgenic mice. *Neurobiology of Disease*.

[11] Dang M. T., Yokoi F., McNaught K. S. P. (2005). Generation and characterization of Dyt1 ΔGAG knock-in mouse as a model for early-onset dystonia. *Experimental Neurology*.

[12] Pandey U. B., Nichols C. D. (2011). Human disease models in *Drosophila melanogaster* and the role of the fly in therapeutic drug discovery. *Pharmacological Reviews*.

[13] Kim A.-Y., Seo J. B., Kim W.-T. (2015). The pathogenic human Torsin A in *Drosophila* activates the unfolded protein response and increases susceptibility to oxidative stress. *BMC Genomics*.

[14] Matyas F., Sreenivasan V., Marbach F. (2010). Motor control by sensory cortex. *Science*.

[15] Dion P. A., Daoud H., Rouleau G. A. (2009). Genetics of motor neuron disorders: new insights into pathogenic mechanisms. *Nature Reviews Genetics*.

[16] Franceschini A., Szklarczyk D., Frankild S. (2013). STRING v9.1: protein-protein interaction networks, with increased coverage and integration. *Nucleic Acids Research*.

[17] Szklarczyk D., Franceschini A., Kuhn M. (2011). The STRING database in 2011: functional interaction networks of proteins, globally integrated and scored. *Nucleic Acids Research*.

[18] Lee Y. S., Carthew R. W. (2003). Making a better RNAi vector for *Drosophila*: use of intron spacers. *Methods*.

[19] Lee D.-W., Seo J. B., Ganetzky B., Koh Y.-H. (2009). ΔFY mutation in human torsina induces locomotor disability and abberant synaptic structures in *Drosophila*. *Molecules and Cells*.

[20] Steentoft C., Vakhrushev S. Y., Joshi H. J. (2013). Precision mapping of the human O-GalNAc glycoproteome through Simple Cell technology. *The EMBO Journal*.

[21] Julenius K. (2007). NetCGlyc 1.0: prediction of mammalian C-mannosylation sites. *Glycobiology*.

[22] Mi H., Dong Q., Muruganujan A., Gaudet P., Lewis S., Thomas P. D. (2009). PANTHER version 7: improved phylogenetic trees, orthologs and collaboration with the Gene Ontology Consortium. *Nucleic Acids Research*.

[23] Zhang Y. Q., Friedman D. B., Wang Z. (2005). Protein expression profiling of the *Drosophila* fragile X mutant brain reveals up-regulation of monoamine synthesis. *Molecular & Cellular Proteomics*.

[24] Muraro N. I., Moffat K. G. (2006). Down-regulation of torp4a, encoding the *Drosophila* homologue of torsinA, results in increased neuronal degeneration. *Journal of Neurobiology*.

[25] Wakabayashi-Ito N., Dohert O. M., Moriyama H. (2011). *dtorsin*, the *Drosophila* ortholog of the early-onset dystonia *TOR1A* (*DYT1*), plays a novel role in dopamine metabolism. *PLoS ONE*.

[26] Bassell G. J., Warren S. T. (2008). Fragile X syndrome: loss of local mRNA regulation alters synaptic development and function. *Neuron*.

[27] Rabilloud T., Lelong C. (2011). Two-dimensional gel electrophoresis in proteomics: a tutorial. *Journal of Proteomics*.

[28] Gonzàlez-Duarte R., Albalat R. (2005). Merging protein, gene and genomic data: the evolution of the MDR-ADH family. *Heredity*.

[29] Cai H., Cong W. N., Ji S., Rothman S., Maudsley S., Martin B. (2012). Metabolic dysfunction in Alzheimer's disease and related neurodegenerative disorders. *Current Alzheimer Research*.

[30] Dunn L., Allen G. F., Mamais A. (2014). Dysregulation of glucose metabolism is an early event in sporadic Parkinson's disease. *Neurobiology of Aging*.

[31] Lemmens R., Melissa M. J., Al-Chalabi A., Brown A. R. H., Robberecht W. (2010). RNA metabolism and the pathogenesis of motor neuron diseases. *Trends in Neurosciences*.

[32] Su L., Cai Y., Xu Y., Dutt A., Shi S., Bramon E. (2014). Cerebral metabolism in major depressive disorder: a voxel-based meta-analysis of positron emission tomography studies. *BMC Psychiatry*.

[33] Carbon M., Su S., Dhawan V., Raymond D., Bressman S., Eidelberg D. (2004). Regional metabolism in primary torsion dystonia. *Neurology*.

[34] Kim J.-I., Ganesan S., Luo S. X. (1126). Aldehyde dehydrogenase 1a1 mediates a GABA synthesis pathway in midbrain dopaminergic neurons. *Science*.

[35] Hallett M. (2011). Neurophysiology of dystonia: the role of inhibition. *Neurobiology of Disease*.

