# Supplementary material for: Drosophila Torsin Protein Regulates Motor Control and Stress Sensitivity and Forms a Complex with Fragile-X Mental Retardation Protein
Source: Neural Plast. 2016 May 30;2016:6762086. doi: 10.1155/2016/6762086 (PMC4904285; doi:10.1155/2016/6762086)

Supplementary Figure 1. N-glycosylation in DTor was predicted using the NetNGly 1.0 server. Asn 96 in DTor was predicted to be N-glycosylated.

**
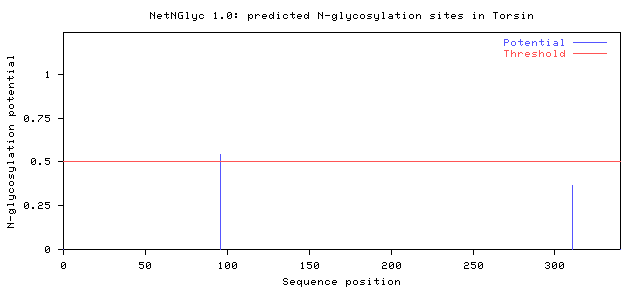
**

Supplementary Figure 2. Control experiments for co-immunoprecipitations without primary antibodies. No DTor, DFMRP, HTor1A, and FMR1 bands were detected from Co-IPs when primary antibodies were not applied.


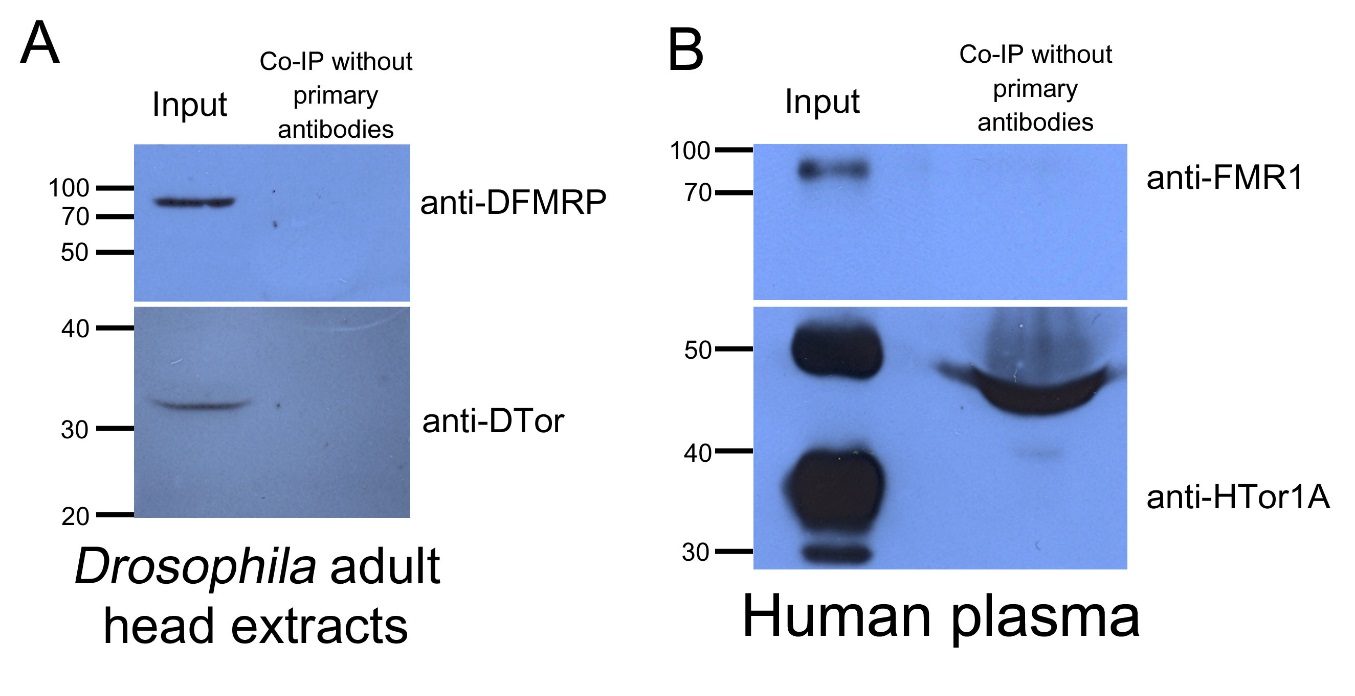

Supplement: Supplementary file 1 — Prediction of Glycosylation in DTor. [file 6762086.f1.zip › 6762086.f1.docx]
