# Supplementary material for: Drosophila Torsin Protein Regulates Motor Control and Stress Sensitivity and Forms a Complex with Fragile-X Mental Retardation Protein
Source: Neural Plast. 2016 May 30;2016:6762086. doi: 10.1155/2016/6762086 (PMC4904285; doi:10.1155/2016/6762086)
Supplement: Supplementary file 1 — Prediction of Glycosylation in DTor. [file 6762086.f1.zip › description.docx]

Supplementary Materials and Methods

**Prediction of glycosylation in DTor**

The possible glycosylation patterns of DTor proteins were predicted using NetNGlyc 1.0 server (<http://www.cbs.dtu.dk/services/NetNGlyc/>) for N-glycosylation, the NetOGlyc 4.0 server (http://www.cbs.dtu.아 /services/NetOGlyc/) for O-glycosylation [19], and the NetCGlyc 1.0 sever (<http://www.cbs.dtu.dk/services/NetCGlyc/>) for C-glycosylation [20]. Only a single asparagine residue in DTor was predicted to be N-glycosylated (Supplementary Figure 1).

**Co-immunoprecipitation**

To examine whether Torsin and FMRP were present in the same protein complexes, rabbit anti-DTor antibodies, rabbit anti-HTor1A antibodies [6, 18], mouse anti-DFMRP antibodies, and mouse anti-FMR1 antibodies were used for co-immunoprecipitation. Protein extracts from adult heads or larval body wall muscle preparations with brains were pre-cleared with normal rabbit or mouse IgG agarose beads (Santa Cruz Biotech., Santa Cruz, CA, USA) and protein A/G plus agarose beads (Santa Cruz Biotech.). After mixing with 20 μl mouse anti-DFMRP antibodies at 4°C overnight, protein A/G agarose beads pre-blocked with 1% bovine serum albumin (Amresco Inc., Solon, OH, USA) in RIPA buffer were added. We also performed control experiments without primary antibodies. After incubation at 4°C for 2 hr with shaking, agarose beads were precipitated by centrifuging them at 15,000 rpm for 10 minutes. After the beads were washed 3 times with RIPA buffer, sample buffers were added, and the samples were boiled before being loaded on a 12% SDS PAGE gel. Proteins were transferred onto a nitrocellulose membrane and then probed with appropriate antibodies.
